# Isolated central nervous system relapse in acute myeloid leukemia: a case report and review of therapeutic challenges

**DOI:** 10.3389/fonc.2025.1667681

**Published:** 2025-10-20

**Authors:** Colin Burns, Ian Muir, Jennifer Foster, Anthony Emanuel, Mark Kochenderfer

**Affiliations:** ^1^ Virginia Tech Carillion School of Medicine, Roanoke, VA, United States; ^2^ Department of Internal Medicine, Carillion Clinic, Roanoke, VA, United States; ^3^ Blue Ridge Cancer Care, Roanoke, VA, United States; ^4^ Department of Pathology, Carilion Clinic, Roanoke, VA, United States

**Keywords:** acute myeloid leukemia, isolated CNS relapse, intrathecal chemotherapy, leptomeningeal disease, CEBPA mutation

## Abstract

Central nervous system (CNS) relapse in acute myeloid leukemia (AML) is an uncommon but clinically significant event, with isolated CNS involvement occurring in a minority of cases and often eluding standard surveillance protocols. We report the case of a 60-year-old man with biallelic CEBPA-mutated AML and complex cytogenetics who achieved two complete remissions over four years before developing isolated leptomeningeal relapse involving the cauda equina. Despite a favorable molecular profile, CSF analysis revealed more than 3,000 WBCs with 97% blasts in the absence of marrow disease. The patient was treated with intrathecal methotrexate, cytarabine, and hydrocortisone, and later transitioned to an Ommaya reservoir. His response was complicated by persistent neurologic deficits and treatment-related neurotoxicity, culminating in functional decline, disease progression in the CNS, and death under hospice care. This case underscores the diagnostic and therapeutic challenges of isolated CNS recurrence in AML, including limited intrathecal drug delivery to nerve roots, the lack of CSF molecular profiling, and the potential for clonal evolution. Given the poor prognosis and therapeutic resistance associated with such cases, our findings support the consideration of CSF surveillance and combined systemic-intrathecal therapy in high-risk patients, particularly those with monocytic subtypes, elevated LDH, or complex cytogenetics.

## Introduction

Acute Myeloid Leukemia (AML) is a malignant clonal disorder arising from myeloid progenitor cells, accounting for approximately 1% of all new cancer diagnoses and representing the most common form of acute leukemia in adults (1). Central nervous system (CNS) involvement in AML, though rare at diagnosis, is associated with poor prognosis and is more frequently observed at relapse. CNS infiltration can present as leptomeningeal disease, cranial nerve palsies, or rarely, parenchymal masses (2).

In contrast to Acute Lymphoblastic Leukemia (ALL), CNS relapse in AML is uncommon, with an estimated incidence ranging from 1% to 4% (3–6). This may be underestimated due to the absence of routine diagnostic lumbar punctures in asymptomatic adult patients. Several studies have demonstrated that routine cerebrospinal fluid (CSF) surveillance does not improve detection rates, supporting current guidelines that do not recommend CNS prophylaxis in AML.

Risk factors for CNS involvement include core-binding factor translocations, FLT3-ITD mutations, younger age (<64 years), leukocytosis at diagnosis (WBC >32 ×10^9^/L), monocytic subtypes, and elevated serum LDH (7–9). Other proposed risk factors from isolated studies include KMT2A mutations, trisomy 8, and concurrent extramedullary disease.

The median time to CNS relapse is seven months, compared to nine months in patients with isolated bone marrow relapse (10). In some studies, 5-year disease-free survival and overall survival are shorter in AML patients with CNS involvement compared to those without CNS involvement (18% *vs* 50% and 19% *vs*. 46%, respectively) (11). However, in other studies, there was no significant difference in median overall survival between AML patients with or without CNS involvement (3).

AML infiltration into the CNS can be difficult to treat with systemic chemotherapy due to the impermeability of the blood-brain barrier. However, certain systemic regimens, including high-dose cytarabine (HD Ara-C) have shown efficacy in treating CNS disease. In addition, adhesion molecules on the blasts improve AML evasion of CNS-directed therapies (12). Intrathecal Methotrexate and Cytarabine with or without hydrocortisone (aka triple therapy), delivered via a lumbar puncture (LP) or Ommaya reservoir, is a commonly preferred first-line option in the setting of AML with CNS involvement (12, 13). Due to AML’s relatively low incidence rate of CNS relapse, CNS-directed prophylaxis is not standard. HD Ara-C induction therapy or high-dose methotrexate have both been found to reduce the tumor load and can be used as adjuncts to intrathecal treatment (14, 15). However, the rate of CNS or independent bone marrow recurrence with either of these systemic chemotherapies is high (16).

Zheng et al. defined isolated CNS relapse as CSF positivity with a concurrent negative bone marrow biopsy within 30 days. Studies have mostly grouped CNS involvement into leptomeningeal and cranial nerve categories. Zheng adds another classification of myeloid sarcoma and argues for the use of radiotherapy. Intrathecal therapy is the standard for both leptomeningeal and myeloid sarcoma; however, there are questions regarding the efficacy of intrathecal agents in isolated cranial nerve involvement. The theory is that cranial nerve palsies develop due to increased pressures in a confined anatomical space and that the CSF will not reach distal portions of the neurovascular apparatus. Thus, it may be more prudent in such a situation to administer systemic chemotherapy, which will be delivered to the target area more effectively. Zheng even shows four of six patients achieving clinical remission with systemic HD-Ara-C (8).

## Case description

The patient was 60 years old at the time of initial presentation to our emergency department in 2020. His past medical history was notable for prostate cancer status post prostatectomy four years prior. His complaint of right knee pain and swelling led to the diagnosis of right lower extremity deep vein thrombosis (DVT). He was started on a direct oral anticoagulant and was discharged home. However, he returned 3 days later with increased swelling of the same leg, and CT imaging was obtained that demonstrated borderline enlarged periportal & pericaval lymph nodes. He was started on antibiotics for suspected infected DVT and admitted. Overnight, he became hypotensive and required an upgrade to the intensive care unit for vasopressor administration. A blood count with differential noted blasts, prompting a peripheral smear which showed Auer rods. He spontaneously developed tumor lysis syndrome and was started on urate-lowering therapy. Bone marrow was obtained, demonstrating the following results:

- Variable cellularity with 80% blasts
- Flow cytometry:
  - Strong expression of CD11b, CD11c, CD38, CD64, and HLA-DR
  - Subsets of CD13, CD14, and CD16
  - Expanded CD45 dim extending into the monocyte region
- Fluorescence *in situ* hybridization:
  - Negative for FLT3, CBFB, MLL, PML/RARa, and RUNX1/RUNX1T1
- Karyotyping:
  - 46,XY, del(9)(q13q22) [2], 46,XY [18]
- Next-generation sequencing (NGS):
  - Biallelic mutation of CEBPA

He was started on induction chemotherapy with cytarabine plus daunorubicin, which was complicated by febrile neutropenia due to gram-negative bacteremia from a contaminated central line, as well as mucositis, ileus, and left buccal thrombophlebitis/cellulitis. A repeat bone marrow biopsy after 4 weeks of therapy showed no evidence of disease. The patient was then started on consolidation therapy with the same agents and remained in remission for the next 18 months after turning down evaluation for allogeneic stem cell transplantation.

At an appointment for routine surveillance in October 2021, he was found to be neutropenic with an absolute neutrophil count of 0.76 K/µL. This prompted a repeat bone marrow biopsy, which revealed 76% blasts. His immunophenotype was unchanged, but his karyotype was noted to be complex with slight variation in the 9q deletion: 46,XY,del(9)(q12q32) [5]/46,XY [15]. Repeat induction chemotherapy with fludarabine, cytarabine, idarubicin, and venetoclax followed by stem cell transplant was recommended, but the patient was concerned about becoming debilitated given poor tolerance of the previous induction therapy. Instead, he opted for outpatient therapy with venetoclax plus decitabine as either palliative intent or as a potential bridge to transplant, although he ultimately decided against transplantation. A bone marrow biopsy was negative for morphological and immunophenotype evidence of AML after 14 weeks of treatment.

From February 2022 to September 2024, the patient remained on venetoclax plus decitabine with a negative bone marrow in October 2023. The only complication during this period was hospitalization for bilateral pulmonary emboli.

**Figure 1:**
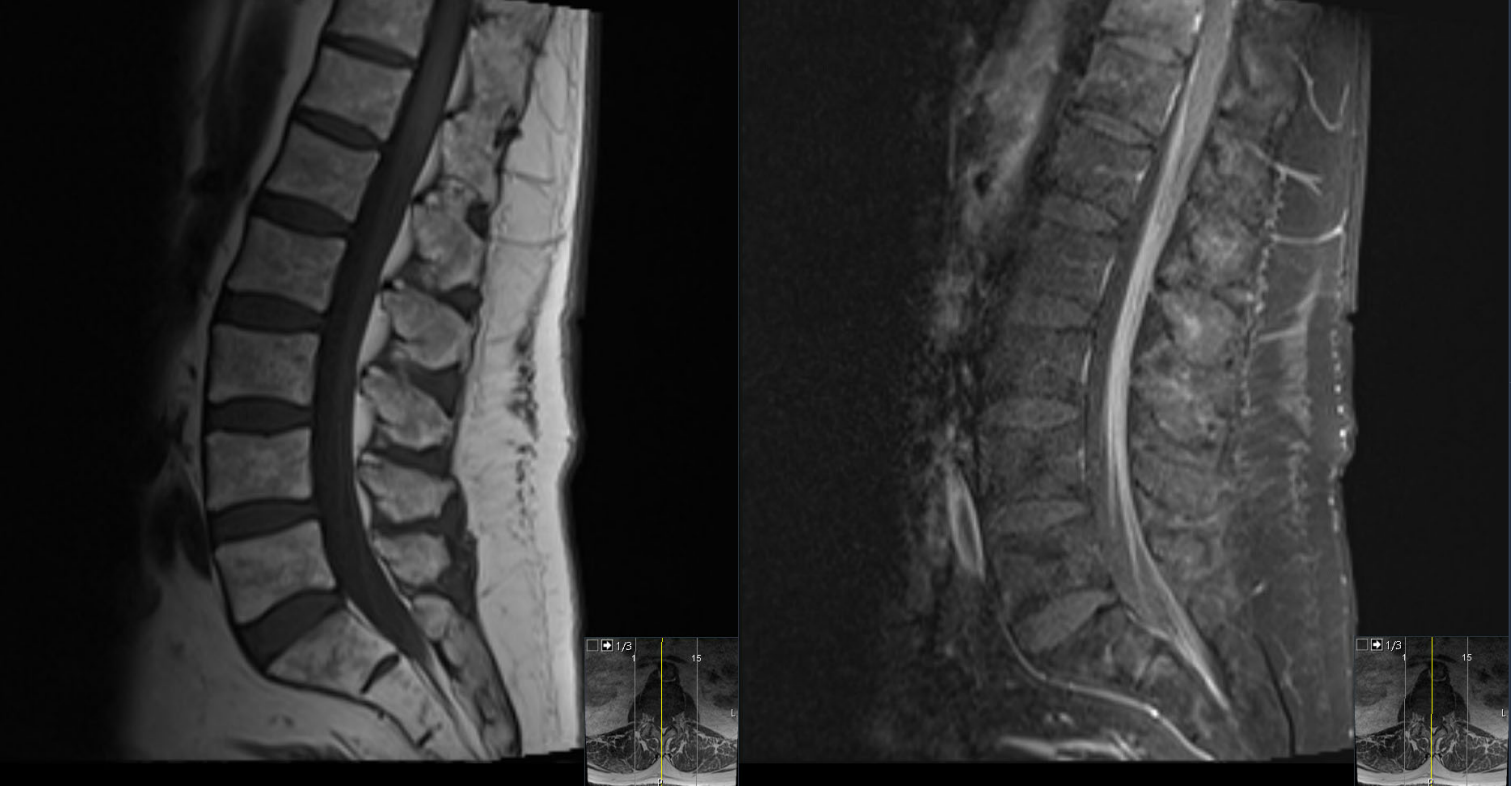
T1-weighted midsagittal lumbar spine MRI with precontrast (left) and postcontrast (right) images showing diffuse enhancement of the cauda equina rootlets.

**Figure 2:**
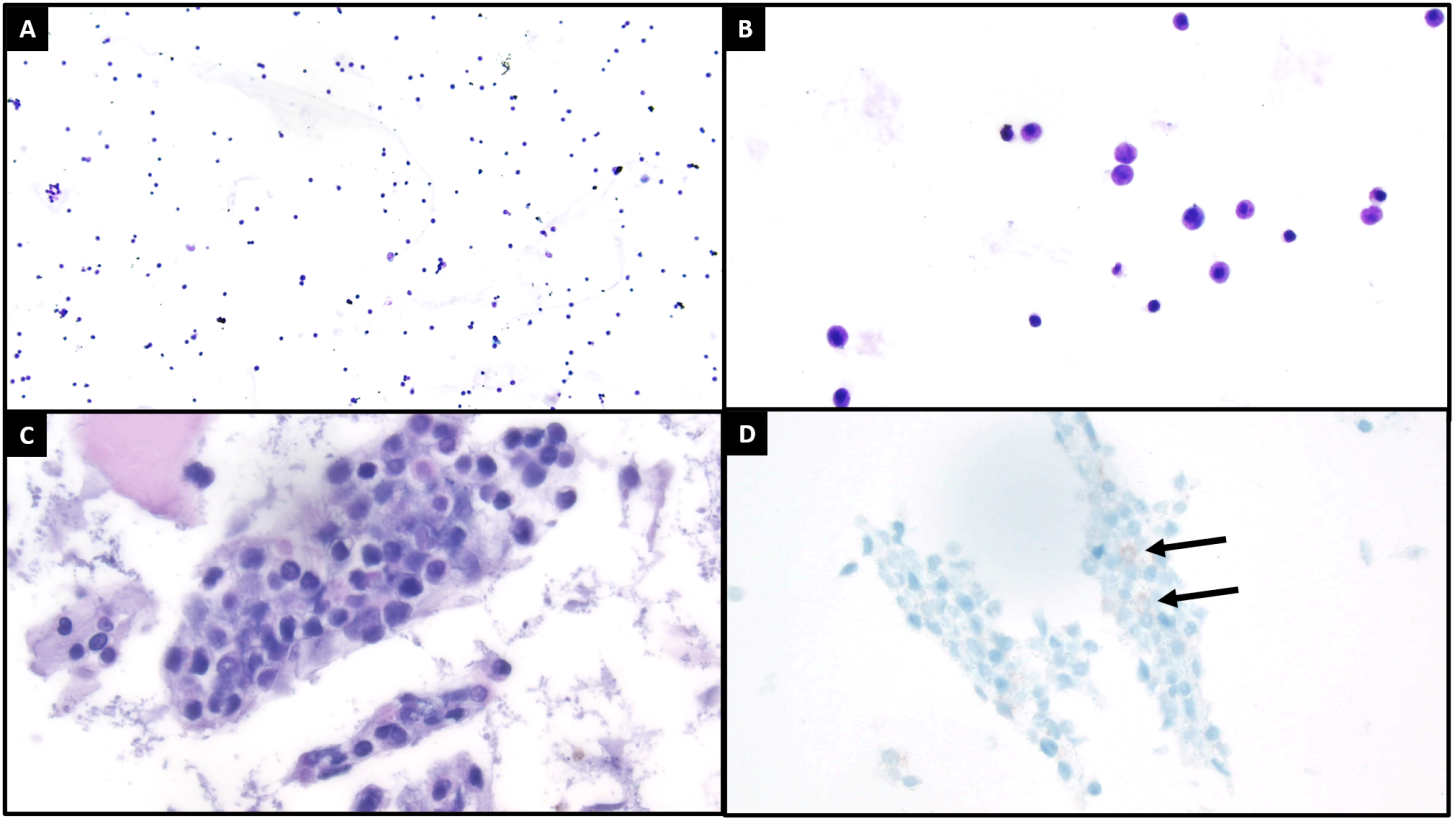
Cytologic evaluation of the cerebrospinal fluid demonstrated increased cellularity compared to what is typically seen [**(A)**, ThinPrep, 100x]. On higher power [**(B)**, ThinPrep, 400x], the cells of interest are characterized by enlarged size, high nuclear to cytoplasmic ratios, occasionally visible nucleoli, and open chromatin patterns. The cell block [**(C)**, H&E, 400x] recovered small clusters of these cells of interest, with a similar cytomorphologic appearance as previously described. Both the ThinPrep and cell block H&E preparations also exhibited background degenerating changes and debris. A CD117 immunohistochemical stain [**(D)**, 400x] demonstrated focal positivity in the cells of interest, with suboptimal staining likely relating to degenerative artifact. The immunophenotype of these cells was better assessed by concurrent flow cytometry (see flow **Figure 3**).

**Figure 3:**
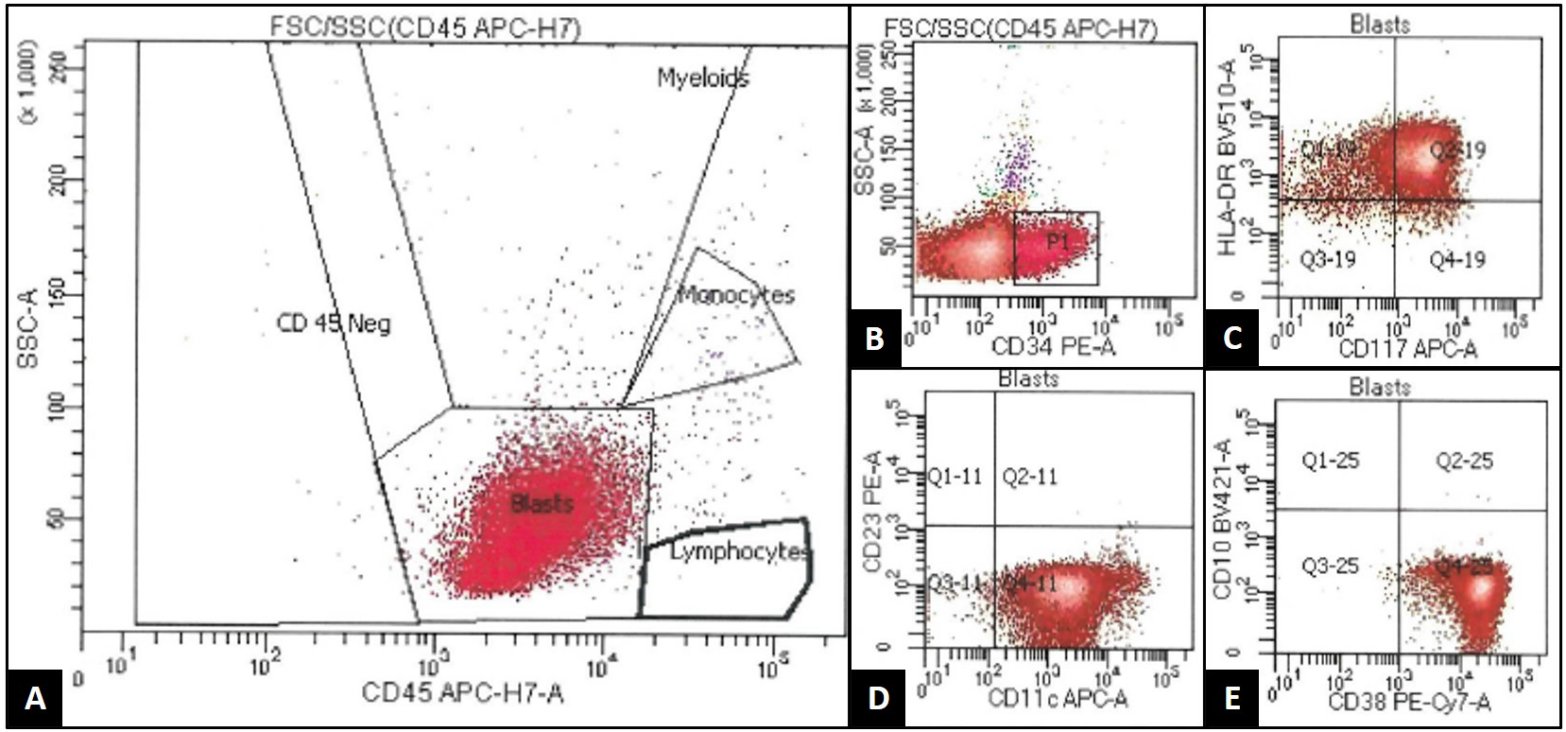
8-color flow cytometry (FACSCanto, BD BioSciences) of the patient's cerebrospinal fluid (CSF) demonstrates an expanded population of events occurring within the "blast gate," characterized by low side scatter and dim CD45 expression **(A)**. This population was enlarged in size by forward scatter property (not pictured), and it also demonstrated expression of CD34 [subset/spectrum; **(B)**], CD117 **(C)**, HLA-DR **(C)**, CD11c **(D)**, and CD38 **(E)**. This population was negative for other B-cell, T-cell, and monocytic markers not listed. This immunophenotype was similar to that seen previously in the patient's original blast population, supporting CSF recurrence of acute myeloid leukemia.

At the end of this period, the patient presented to multiple providers with complaints of generalized weakness and fatigue. These symptoms were accompanied by intermittent hearing difficulties/ear fullness/tinnitus as well as periods of left lower extremity weakness. A referral to audiology revealed no hearing loss. In the interim, he developed fevers, worsening headaches, and burning neuropathic back pain that radiated down both legs. He sought emergency medical treatment, where he was noted to have ⅘ strength throughout bilateral lower extremities accompanied by areflexia, but no sensory deficits. Imaging was notable for extensive cauda equina enhancement without vertebral involvement (see **Figure 1**), suspicious for Guillan-Barre Syndrome. Lumbar puncture revealed >3000 WBCs with 97% CD34+ myeloblasts (see **Table 1**), elevated protein, and decreased glucose. Molecular studies were sent, but were difficult to analyze given the small quantity of cells and a low mitotic index. Ultimately, both the cytogenetic and immunophenotypic results of the CSF (see **Figures 2**, **3**) were unchanged from those found in the marrow during the first relapse.

**Table 1:** Timeline of cerebrospinal fluid cytology results from the time of diagnosis through the duration of intrathecal chemotherapy. White blood cell (WBC) counts and blast percentages are shown.

Date	WBC (cells)/mL	Blasts (%)	Treatment	Other
10/3	3232	97	No	
10/5	5009	96	Yes	
10/8	1384	76	Yes	
10/11	57	42	Yes	
10/14	26	72	Yes	
10/17	2	41	Yes	
10/24	36	76	Yes	
10/31	17	70	Yes	
11/5	n/a	n/a	Yes	No fluid samples sent
11/7	31	90	Yes	

He was started on levetiracetam for seizure prophylaxis, and IR was consulted for delivery of twice weekly intrathecal methotrexate, cytarabine, and hydrocortisone until CNS clearance. A bone marrow biopsy was obtained 3 days later and showed absence of morphological and immunophenotypic evidence of disease. Serial CSF studies demonstrated the following:

The patient initially showed a reduction in WBC and blast percentages. He progressively recovered strength in his bilateral lower extremities, however, his auditory symptoms persisted. The patient unfortunately did not tolerate the treatments well, suffering from excruciating back pain despite escalating doses of opioid therapy. An Ommaya reservoir was placed but resulted in intractable vomiting. He required longer recovery periods, only receiving treatment once weekly, and started to demonstrate a gradual functional decline. Weakness returned to his right lower extremity as well as diffuse radiculoneuritis. Subsequent intrathecal treatment was given; however, studies revealed failure to clear the CSF after nine treatments and he became progressively more debilitated. He ultimately transitioned to a comfort-focused treatment plan and passed away shortly after returning home with hospice.

## Discussion

Isolated CNS relapse in AML is a rare, but clinically significant phenomenon (2). CNS relapse is most often observed in the context of concurrent systemic disease, with isolated CNS recurrence accounting for a minority of cases. Zheng et al. documented 34 patients out of 432 with isolated CNS recurrence in the setting of AML (8). Isolated CNS relapse may escape detection with standard surveillance and pose unique therapeutic challenges. Our patient had an unusual combination of favorable mutations with complex cytogenetics. His abnormal karyotype was present at diagnosis, yet he responded well to his initial standard induction as well as the palliative regimen at relapse, indicating potentially a more favorable cytogenetic profile due to the presence of biallelic CEPBA. However, similar cytogenetics were noted in the CNS, but the patient failed to mount a similar response to chemotherapy.

Our patient’s clinical course raises several important questions regarding the pathophysiology, treatment response, and appropriate management of CNS relapse in AML. At initial diagnosis, he exhibited a favorable molecular profile with biallelic CEBPA mutations but also showed complex cytogenetic abnormalities, including a del(9q). This may have contributed to the patient’s initial responsiveness to therapy—achieving remission following two separate induction regimens—while also foreshadowing eventual therapeutic resistance. Although biallelic CEBPA mutations are typically associated with a favorable prognosis and high remission rates, this was not reflected in the patient’s response to intrathecal therapy. The poor response may have been due to undetected clonal evolution or cytogenetic progression within the CNS, as no follow-up karyotyping of CSF was performed. Given this possibility, we propose that CSF cytogenetic or molecular profiling should be considered in suspected isolated CNS relapse to guide therapy more effectively.

The variable response to intrathecal therapy in AML with CNS relapse highlights the importance of disease localization within the CNS. Zheng et al. categorized CNS involvement into three morphologic groups: leptomeningeal disease, cranial nerve involvement, and myeloid sarcoma. Our patient’s findings most closely resemble leptomeningeal disease, as involvement of the conus medullaris and cauda equina is commonly categorized under leptomeningeal disease. This distinction matters, as it may impact the efficacy of intrathecal therapy. Cranial and spinal nerves have limited exposure to CSF circulation, and drug penetration into these regions may be inadequate. For example, in the context of cranial nerve involvement, systemic high dose cytarabine has demonstrated better response rates than intrathecal therapy alone (8). It remains unclear whether systemic chemotherapy should routinely be added to intrathecal regimens, but this case and others suggest that it may provide therapeutic benefit.

A similar case by Suárez et al. described a patient with isolated cauda equina relapse of FLT3-ITD and CEBPA-mutated AML who responded well to a multi-modality approach including IT chemotherapy, systemic methotrexate, sorafenib, and craniospinal irradiation, remaining in remission for over three years (2). While such aggressive therapy may not be appropriate for all patients, particularly those with comorbidities or poor performance status, it raises important questions regarding the standard of care in isolated CNS relapse. In our case, it remains unclear whether the patient’s disease was intrinsically resistant to the selected agents, whether drug delivery was inadequate, or whether additional systemic or radiotherapeutic measures might have altered the outcome.

Another consideration is whether certain risk factors that predispose to CNS involvement also correlate with treatment resistance. The patient’s initial diagnosis revealed monocytic differentiation, previously designated FAB M5, a known risk factor for CNS relapse (17). Johnson et al. reported that 45% of all patients with isolated CNS relapse of AML exhibited an FAB M5 classification. This increased risk may be partly explained by the higher prevalence of AML with monocytic differentiation in a younger patient population, who are predisposed to CNS involvement due to greater leptomeningeal vascularity and a higher rate of extramedullary leukemia (18). Other factors, such as complex karyotype, elevated LDH, and extramedullary involvement, have also been implicated in both CNS infiltration and therapeutic resistance (7, 9). More research is needed to identify high-risk molecular or clinical signatures that can better stratify patients for CNS surveillance and tailored therapy, as well as to assess whether the type and location of CNS involvement correlate with refractory disease.

Treatment-related neurotoxicity was a major complicating factor in our patient’s course. Although intrathecal chemotherapy is generally well-tolerated, especially in pediatric populations, adult data are limited. A single-center study at MD Anderson Cancer Center described several cases of adults with leukemia or lymphoma treated with methotrexate plus cytarabine, who developed myelopathy presenting as polyneuropathy or polyradiculopathy. The most prevalent findings among them were dorsal column enhancement on T2-weighted MRI. Interestingly, enhancement was also seen in two patients along the cauda equina nerve roots (19). There is some evidence that this occurs mostly with simultaneous systemic administration (7). Neurotoxicity is particularly challenging to distinguish from leukemic infiltration. In our case, the patient initially experienced partial neurologic recovery, followed by clinical decline and rising blast counts in the CSF. Whether this represented progression of disease, treatment toxicity, or both remains unclear. No follow-up imaging was obtained to aid in etiological determination.

The disappointing outcome in this case highlights broader challenges in the management of isolated CNS involvement in AML. While intrathecal chemotherapy remains the cornerstone of treatment, its limitations—particularly in cases of nerve root involvement—are increasingly recognized. Combining systemic chemotherapy with intrathecal therapy may offer improved outcomes, particularly when extramedullary spread is suspected or confirmed. However, this must be balanced against the potential for increased toxicity, especially in older or frail patients. In our patient’s case, the cumulative burden of symptoms and adverse effects contributed to the decision to pursue comfort-focused care.

Finally, the presented case prompts reconsideration of current surveillance strategies in AML. Specifically, regarding whether CSF analysis should be incorporated alongside bone marrow biopsies. It is our belief that CSF sampling in the absence of neurological symptoms does not significantly change the detection rate of CNS involvement. However, given the poor prognosis associated with delayed detection, selective CSF evaluation may be warranted in patients with high-risk features such as FAB M4/M5 subtypes, WBC greater than 100 x 10^9^/L, elevated LDH, prior CNS involvement, and 11q abnormalities.

## Data Availability

The original contributions presented in the study are included in the article/supplementary material. Further inquiries can be directed to the corresponding author.
